# Thermally Stable and Energy Efficient Newly Synthesized Bipolar Emitters for Yellow and Green OLED Devices

**DOI:** 10.3390/molecules31010158

**Published:** 2026-01-01

**Authors:** Anil Kumar, Sushanta Lenka, Kapil Patidar, Chih-An Tung, Ming Yu Luo, Raminta Beresneviciute, Gintare Krucaite, Daiva Tavgeniene, Dovydas Blazevicius, Bernadeta Blazeviciute, Jwo-Huei Jou, Saulius Grigalevicius

**Affiliations:** 1 Department of Materials Science and Engineering, National Tsing Hua University, No. 101, Section 2, Guangfu Rd., East District, Hsinchu 30013, Taiwan; anilgpchkee@gmail.com (A.K.);; 2 Department of Polymer Chemistry and Technology, Kaunas University of Technology, Radvilenu Plentas 19, LT50254 Kaunas, Lithuaniadovydas.blazevicius@ktu.lt (D.B.);

**Keywords:** bipolar emitter, thermal stability, glass transition temperature, organic light-emitting diode, efficiency, photo-therapeutic

## Abstract

Organic light-emitting diodes (OLEDs) have emerged as a leading high-resolution display and lighting technology, as well as for photo-therapeutic applications, due to their light weight, flexibility, and excellent color rendering. However, achieving long-term thermal stability and high energy efficiency remains a principal issue for their widespread adoption. Strong thermal robustness in OLED emitter materials is a critical parameter for achieving long device lifetimes, stable film morphology, reliable high-temperature processing, and sustained interface integrity in high-performance hosts. Bipolar emitters RB14 (*N*-(9-ethylcarbazole-3-yl)-4-(diphenylamino)phenyl-9*H*-carbazole-9-yl-1,8-naphthalimide), RB18 (*N*-phenyl-4-(diphenylamino)phenyl-9*H*-carbazole-9-yl-1,8-naphthalimide), and RB22 (*N*-phenyl-3-(2-methoxypyridin-3-yl)-9*H*-carbazole-9-yl-1,8-naphthalimide) were newly synthesized. RB18 is a yellow bipolar OLED emitter that has a glass transition temperature (T_g_) of 162 °C and thermal durability (T_d_) of 431 °C, which is the highest reported value for naphthalimide-based bipolar emitter derivatives for yellow OLEDs. Meanwhile, RB14 and RB22 are green OLED emitters that have glass transition temperatures (T_g_) of 133 °C and 167 °C, and thermal durabilities (T_d_) of 336 °C and 400 °C, respectively. We have fabricated OLED devices using these bipolar emitters dispersed in CBP host matrix, and we have found that the maximum EQEs (%) for RB14, RB18, and RB22 emitter-based devices are 7.93%, 3.40%, and 4.02%, respectively. For confirmation of thermal stability, we also used UV-visible spectroscopy measurements at variable temperatures on annealed spin-coated glass films of these emitter materials and found that RB22 is the most thermally stable emitter among these materials.

## 1. Introduction

Organic light-emitting diodes (OLEDs) have emerged as a revolutionary technology in the fields of display and solid-state lighting [1], as well as in photo-therapeutic, catalysis, energy storage, and health-conscious lighting applications [2,3,4], owing to their transparency, flexibility [5], light weight [6], high contrast ratios, wide viewing angles [7], excellent color rendering [8], fast response time, low power [9], and potential for energy-efficient operation [10]. Low-cost fabrication processes, compatible with both vacuum- and solution-based techniques, also support their scalability for large-area production [11,12,13]. Performance lies in the emitting layer because it is the heart of the OLED, which directly influences not only the color purity and brightness, but also the power efficiency and operational lifetime of the device. In practical applications, OLEDs are often exposed to elevated temperatures either due to external environmental conditions or internal joule heating during high-brightness operation [14]. One of the critical factors determining the long-term functionality of OLEDs is the thermal stability of these emitter materials. Thermally unstable emitter materials can undergo morphological changes such as phase separation, crystallization, or molecular aggregation, all of which can lead to device degradation or complete failure [15].

Increasing the glass transition temperature (T_g_) of organic materials could reduce heat-induced morphological changes, hence strengthening the stability of device performance. The glass transition temperature (T_g_) and thermal decomposition temperature (T_d_) of organic materials are key indicators of their robustness under thermal stress [16]. Thermal stability is particularly important in high-performance OLEDs that operate at high luminance, such as in large-area display applications [17]. To meet these demands, recent studies have focused on molecular design strategies that improve morphological and thermal robustness. In particular, bipolar emitter structures containing both electron-donating and electron-accepting groups can facilitate balanced charge transport, minimize charge accumulation, and enhance operational stability and efficiency. Several carbazole-, bicarbazole-, and naphthalimide-based bipolar derivatives have previously been reported as electroactive materials for optoelectronic applications [18,19,20,21].

From an energy efficiency standpoint, emitter materials play a pivotal role in determining the external quantum efficiency (EQE) of OLEDs. Efficient exciton harvesting mechanisms, such as those enabled by phosphorescent materials and thermally activated delayed fluorescence (TADF), are instrumental in achieving high internal quantum efficiencies (IQEs), theoretically up to 100% [22,23]. TADF emitters, in particular, exploit reverse intersystem crossing (RISC) to convert non-radiative triplet states into radiative singlet states, significantly enhancing efficiency without relying on heavy metal complexes [24]. However, the operational stability of TADF emitters under thermal stress remains a challenge, as the RISC process itself is thermally activated and may lead to increased exciton–polaron interactions and degradation over time [25].

In this paper, we report newly synthesized bipolar emitter materials: RB14 (*N*-(9-ethylcarbazole-3-yl)-4-(diphenylamino)phenyl-9*H*-carbazole-9-yl-1,8-naphthalimide), RB18 (*N*-phenyl-4-(diphenylamino)phenyl-9*H*-carbazole-9-yl-1,8-naphthalimide), and RB22 (*N*-phenyl-3-(2-methoxypyridin-3-yl)-9*H*-carbazole-9-yl-1,8-naphthalimide). RB18 is a yellow bipolar OLED emitter that has a glass transition temperature (T_g_) of 162 °C and thermal durability (T_d_) of 431 °C, which are the highest reported values for yellow OLED emitters. In contrast, RB14 and RB22 are green OLED emitters with glass transition temperatures (T_g_) of 133 °C and 167 °C, and thermal durability (T_d_) of 336 °C and 400 °C, respectively. The thermal behavior of these synthesized materials was investigated using thermogravimetric analysis (TGA) and differential scanning calorimetry (DSC) under a nitrogen atmosphere. The thermogravimetric analysis confirmed that the newly synthesized derivatives are highly thermally stable compounds with temperatures of 5% weight loss in the range from 331 °C to 400 °C. We have fabricated OLED devices using these bipolar emitters with a CBP host matrix, and we found that the maximum EQEs (%) for RB14, RB18, and RB22 are 7.93%, 3.40%, and 4.02%, respectively. For the thermal stability validation of these materials, we also analyzed UV-visible spectra at variable temperatures of the films of the spin-coated emitters and found that the RB22 absorbance peak variation is negligible, which confirmed the highest thermal stability of RB22 among these emitting materials.

## 2. Results and Discussion

**Figure 1:**
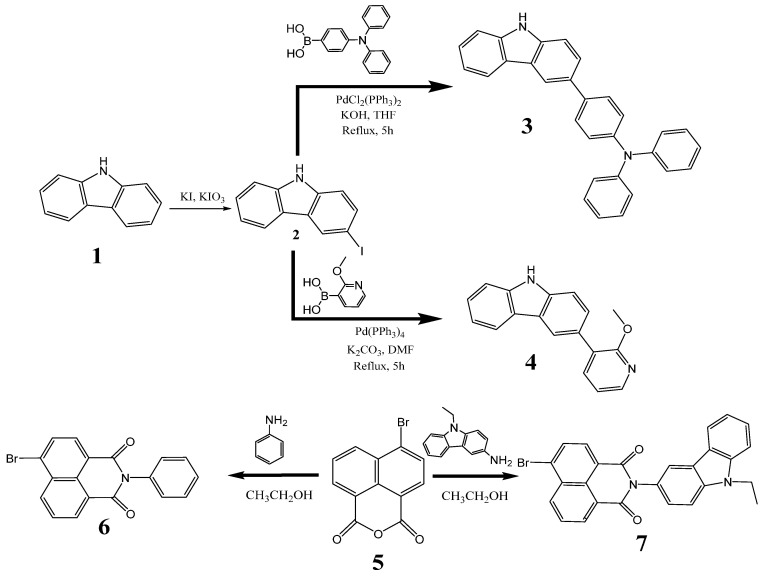
Synthetic pathway for preparation of the key starting compounds **2**–**7**.

Intermediate materials were synthesized through a synthetic route as illustrated in Figure 1. Initially, 3-iodo-9*H*-carbazole (**2**) was prepared according to the procedure described by Tucker [26]. Then, carbazole intermediates (**3** and **4**) were prepared via Suzuki cross-coupling of the 3-iodo-9*H*-carbazole (**2**) with an excess of 4-(diphenylamino) phenylboronic acid or 2-methoxy-3-pyridinylboronic acid, respectively, utilizing a palladium catalyst under basic conditions [27]. Other starting derivatives (**6** and **7**) were synthesized by reaction of commercially available 4-bromo-1,8-napthalic anhydride with, respectively, aniline or 3-amino-9-ethylcarbazole.

**Figure 2:**
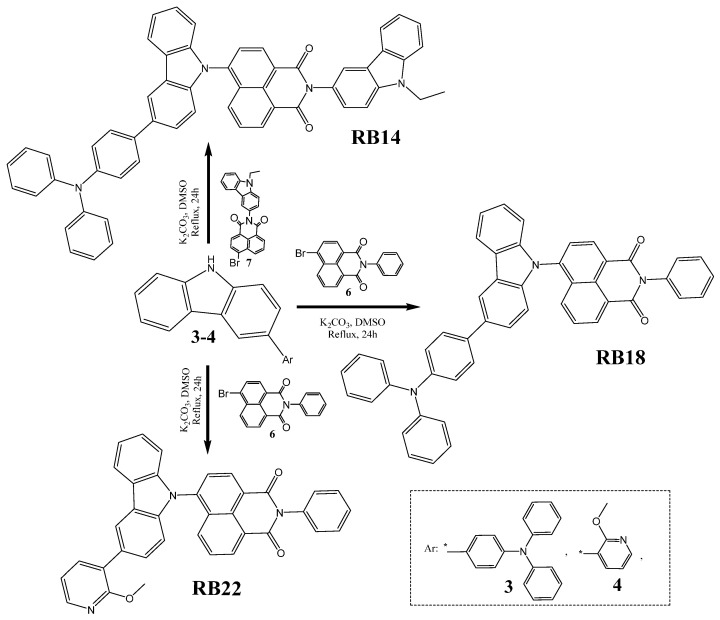
Synthetic pathway of the objective 1,8-naphthalimide-based materials **RB14**, **RB18**, and **RB22**. * represents free radical or an atom or molecule with an unpaired electron.

The objective compound *N*-(9-ethylcarbazole-3-yl)-4-[3-(diphenylamino)phenylcarbazol-9-yl]-1,8-naphthalimide (**RB14**) was synthesized through an Ullmann coupling reaction between *N*-(9-ethylcarbazole-3-yl)-4-bromo-1,8-naphthalimide (**7**) and 3-[4-(diphenylamino)phenyl]-9*H*-carbazole (**3**) in dimethylformamide. The other two target derivatives, *N*-phenyl-4-[3-(diphenylamino)phenylcarbazol-9-yl]-1,8-naphthalimide (**RB18**) and *N*-phenyl-4-[3-(2-methoxypyridin-3-yl)carbazol-9-yl]-1,8-naphthalimide (**RB22**), were also obtained by the Ullmann coupling reaction for the next step between *N*-phenyl-4-bromo-1,8-naphthalimide (**6**) and, respectively, 3-[4-(diphenylamino)phenyl]-9*H*-carbazole (**3**) or 3-(2-methoxypyridin-3-yl)-9*H*-carbazole (**4**). All the detailed synthetic information is provided in the Figure 2 and Supporting Information section of this article.

Mass spectrometry (MS) and nuclear magnetic resonance (NMR) spectroscopy were employed to identify the newly synthesized derivatives. The experimental data supported the proposed structures. All the target compounds demonstrated excellent solubility in common organic solvents, such as chloroform, DMF, and tetrahydrofuran (THF) at room temperature.

### 2.1. Photo-Physical Properties

**Figure 3:**
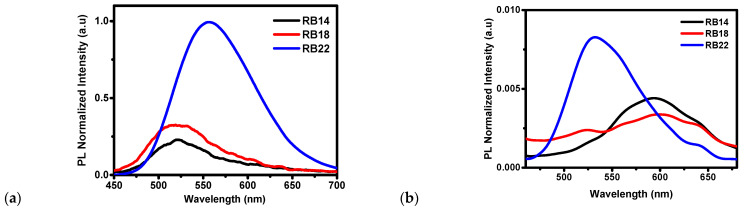
Photoluminescence (PL) spectra of the RB materials (**a**) in THF solvent and (**b**) of thin films.

**Figure 4:**
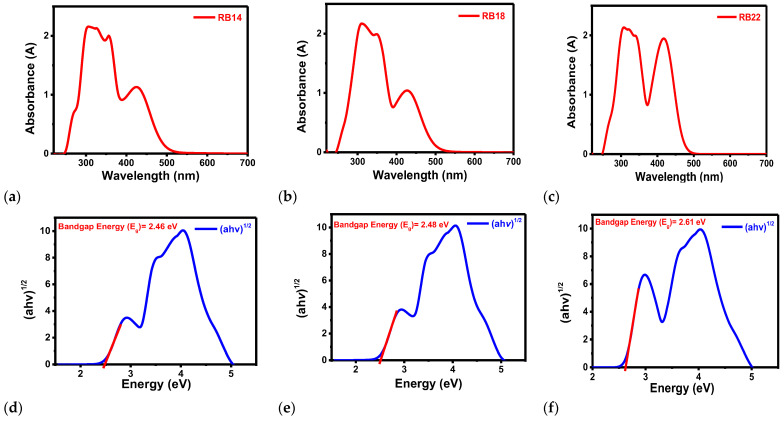
Ultraviolet-visible absorbance (UV-vis abs) spectra, Wavelength vs. Absorbance (A), for compounds: (**a**) **RB14**, (**b**) **RB18**, and (**c**) **RB22**. The respective Tauc plots: (**d**) **RB14**, (**e**) **RB18**, and (**f**) **RB22**, which illustrate the absorption wavelength and bandgap.

The photoluminescence (PL) spectra of compounds **RB14**, **RB18**, and **RB22** are shown in Figure 3a, illustrating peak emission wavelengths of 525 nm, 520 nm, and 555 nm for **RB14**, **RB18**, and **RB22**, respectively, in THF solvent, while the peak emission wavelengths of films on glass shown in Figure 3b were 600 nm, 600 nm and, 535 nm for **RB14**, **RB18**, and **RB22**, respectively. **RB14** has a maximum emission peak at 525 nm in the THF solvent, while at 600 nm over the glass film. **RB18** has a maximum peak emission at 520 nm in the THF solvent, while at 600 nm over the glass film. **RB22** has a maximum emission at 555 nm in the THF solvent, while at 535 nm in the thin film. The maximum peak shift from solution to glass film is higher in **RB14** and **RB18**, from lower to higher wavelength range, while the maximum peak shift from solution to glass film is lower in **RB22**, from higher to lower wavelength range. A red shift indicates that an increase in conjugation length of molecules reduces the electronic band gap. **RB14** and **RB18** exhibit a higher formation of J-aggregates [28]. In **RB22**, the formation of an H-aggregate causes a blue shift [29]. H-aggregated films show that H-type packing restricts exciton delocalization and suppresses non-radiative decay, whereas J-aggregates promote delocalized exciton states that are more vulnerable to quenching and dark-state formation [30]. Blue shift absorption is also responsible for the increase in band gap energy; therefore, **RB22** has the highest band gap energy among these materials, as shown in Figure 4f.

Under ambient conditions, the ultraviolet-visible absorbance (UV-vis abs) spectra of the compounds **RB14**, **RB18**, and **RB22** were examined using THF solvent. Figure 4a–c shows the absorbance spectra of the respective prepared 10 mg/mL solutions measured using a quartz cuvette. A Tauc plot was constructed (Figure 4d–f) using the absorption wavelength and intensity data. The Tauc plot was calculated by employing the following Equation (1) [31]:

(αhν)^2^ = A(hν − E_g_) (1)

where hν is the photon energy (1240/wavelength), α is the absorption coefficient, A is a constant, and E_g_ is the optical bandgap.

It could be observed from the results that the optical band gaps (E_g_) of **RB14**, **RB18**, and **RB22** are 2.46 eV, 2.48 eV, and 2.61 eV, respectively.

### 2.2. Electrochemical Properties

**Figure 5:**
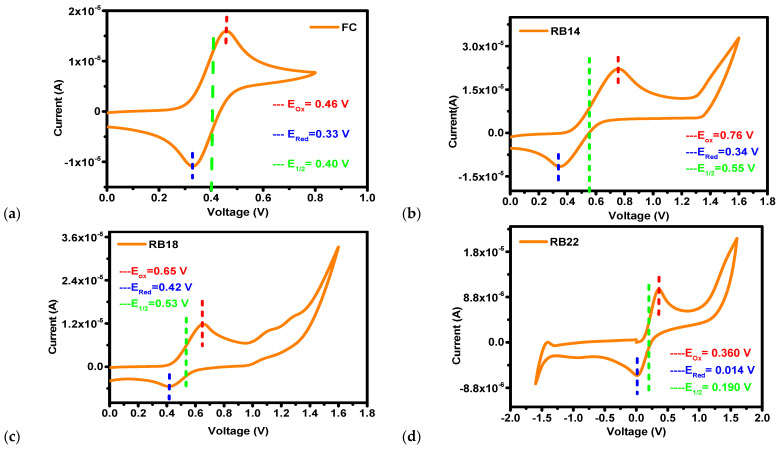
Cyclic voltammetry analysis for calculation of HOMO levels of the compounds (**a**) ferrocene (external standard), (**b**) **RB14**, (**c**) **RB18**, and (**d**) **RB22**.

Cyclic voltammetry (CV) measurements were conducted to estimate the electrochemical characteristics of compounds **RB14**, **RB18**, and **RB22**. The results of these measurements are presented in Figure 5. These results were utilized to calculate highest occupied molecular orbital (HOMO) and lowest unoccupied molecular orbital (LUMO) energy.

Calculation of the HOMO level was performed using Equation (2), and calculation of the LUMO level was performed using Equation (3).

(2) $$E_{HOMO}=-4.8-[\left(E_{ox}-E_{1/2\left(Ferrocene\right)}\right)]$$

(3) $$E_{LUMO}=E_{HOMO}+E_{g}$$

where E_ox_ is the oxidation onset potential of a material, E_1/2 (Ferrocene)_ is the half-wave potential of ferrocene, E_g_ is the bandgap, E_HOMO_ is the HOMO energy, and E_LUMO_ is the LUMO energy [32].

The bandgap (E_g_) was calculated using a Tauc plot, and the values obtained were utilized to determine the LUMO energy. For **RB14**, **RB18**, and **RB22**, the measured HOMO energy levels were determined to be −5.16, −5.05, and −4.76 eV, respectively. Similarly, the LUMO levels were calculated to be −2.68, −2.59, and −2.15 eV for **RB14**, **RB18**, and **RB22**, respectively. These results indicate that the LUMO level of the derivative **RB14** closely matches with LUMO energy of ETL and of the host material. This band matching is beneficial for electron transport, which may be the reason for the highest EQE of 7.93% at 10 wt% doping with CBP host among all these materials.

### 2.3. Thermal Properties

**Figure 6:**
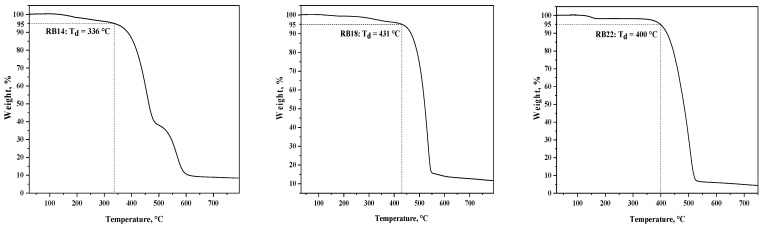
Curves of the TGA measurements of the **RB14**, **RB18**, and **RB22** materials.

**Figure 7:**
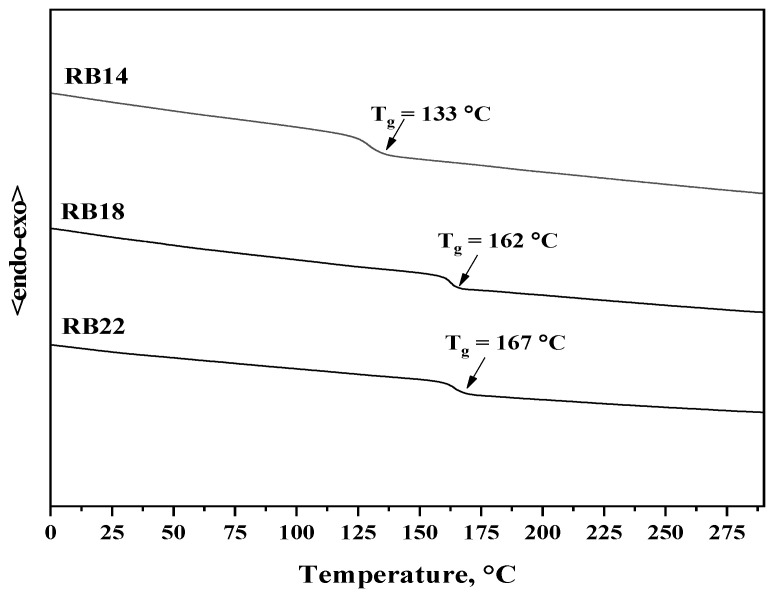
DSC second heating curves of compounds **RB14**, **RB18**, and **RB22**. Heating rate: 10 °C/min.

The thermal behavior of the synthesized materials **RB14**, **RB18**, and **RB22** was investigated using thermogravimetric analysis (TGA), shown in Figure 6, and differential scanning calorimetry (DSC), as shown in Figure 7. The results revealed that the compounds demonstrate high thermal stability. The temperatures of 5% weight loss (T_d_) for derivatives **RB14**, **RB18**, and **RB22** were 336 °C, 431 °C, and 400 °C, respectively, as confirmed by TGA with a heating rate of 10 °C/min.

The derivatives **RB14**, **RB18**, and **RB22** were obtained after synthesis as fully amorphous materials with glass transition temperatures (T_g_) of 133 °C, 162 °C, and 167 °C, respectively, as indicated during the DSC experiment. No crystallization or melting transitions were detected during the first or second heating and cooling cycles. The very high T_g_ values of all target compounds indicate their potential advantage for application in OLEDs. The DSC thermograms of the target compounds **RB14**, **RB18**, and **RB22** during the second heating cycle are presented in Figure 7.

### 2.4. Structure and Characterization of Electroluminescent OLED Devices

**Figure 8:**
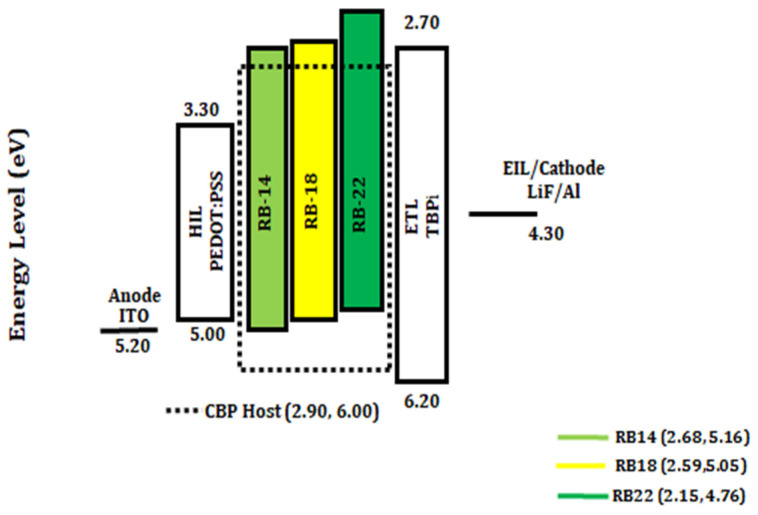
Energy level diagram in eV of the solution-processed yellow and green OLEDs containing host CBP with the emitters **RB14**, **RB18**, and **RB22**.

Preliminary tests of the RB emitting materials in the fabrication and characterization of the OLEDs using host matrix CBP demonstrated that the emitters **RB14**, **RB18**, and **RB22** are promising components of the multilayer devices. The fabricated devices had the structures: ITO (125 nm)/PEDOT: PSS (35 nm)/CBP host: x wt% of RB emitters (x = 5.0, 10, 15, and 20%) (20 nm)/TPBi (40 nm)/LiF (1 nm)/Al (200 nm). The detailed device fabrication process is described in the Experimental part. Figure 8 represents the energy level diagrams of the yellow and green light-emitting OLEDs using the hosts CBP doped with **RB14**, **RB18**, and **RB22** emitters. It was observed that these emitting materials could be suitable for fabricating thermally stable OLEDs.

**Figure 9:**
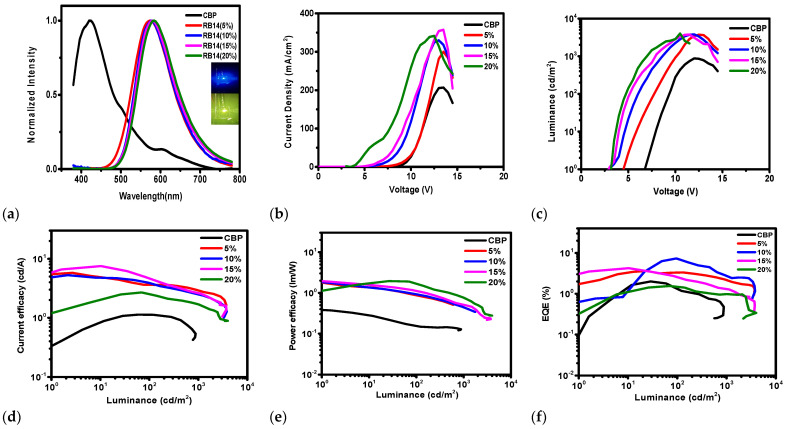
Characteristics of OLEDs using **RB14** emitter of different dopant concentrations in CBP host. (**a**) Electroluminescence spectra, Wavelength vs. Normalized intensity, (**b**) Current density–Voltage, (**c**) Luminance–Voltage, (**d**) Current efficacy–Luminance, (**e**) Power efficacy–Luminance, (**f**) External Quantum Efficiency–Luminance.

The electroluminescent (EL) properties of the devices using the emitter **RB14** at dopant concentrations of 5.0, 10.0, 15.0, and 20.0 wt% with a CBP host were investigated. Figure 9 shows electroluminescence spectra, current density–voltage, luminance–voltage, current efficacy–luminance, power efficacy–luminance, and external quantum efficiency–luminance characteristics of the OLEDs containing the emitter **RB14** with the CBP host. Some characteristics of these devices are also summarized in Table 1. The observed EL spectra (Figure 9a) showed emission peaks for different doping concentrations in the **RB14** emitter and CBP host-based devices. The fabricated devices emitted a yellowish-green light, with CIE coordinates ranging from (0.46, 0.50) to (0.51, 0.48) and broad spectra from 450 to 750 nm with a maximum peak at 578 nm.

The **RB14**-based OLEDs demonstrated driving voltages of 3.6–5.8 V, maximal current efficacies of 2.77–7.39 cd/A, maximal power efficacies of 2.17–2.93 lm/W, maximal external quantum efficiencies of 1.6–7.93% and maximal luminance in the range of 3722–3999 cd/m^2^. At higher brightness, such as 1000 cd/m^2^, which can be used for illumination applications, it was observed that the 10 wt% emitter-based device slightly outperformed the other OLEDs by displaying a maximum power efficacy of 2.4 lm/W, maximum current efficacy of 5.09 and maximal external quantum efficiency of 7.93% with a rather low driving voltage of 4.6 eV, as well as high luminance of about 3800 cd/m^2^. The maximum external quantum efficiency is higher than that of other RB materials because the energy level of **RB14** closely matches the LUMO of the host material and the ETL material.

**Figure 10:**
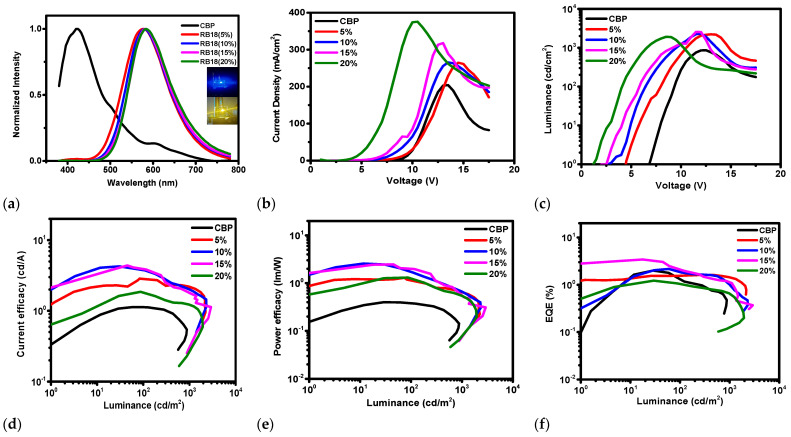
Characteristics of OLEDs using **RB18** emitter of different dopant concentrations with CBP host. (**a**) Electroluminescence spectra, Wavelength vs. Normalized intensity, (**b**) Current density–Voltage, (**c**) Luminance–Voltage, (**d**) Current efficacy–Luminance, (**e**) Power efficacy–Luminance, (**f**) External Quantum Efficiency–Luminance.

The electroluminescent (EL) properties of the devices using the emitter **RB18** at dopant concentrations of 5.0, 10.0, 15.0, and 20.0 wt% with the CBP host were also investigated. Figure 10 shows electroluminescence spectra, current density–voltage, luminance–voltage, current efficacy–luminance, power efficacy–luminance, and external quantum efficiency–luminance characteristics of the OLEDs with the emitter **RB18** dispersed in the CBP host. Some characteristics of these devices are also summarized in Table 2. The observed EL spectra (Figure 10a) showed emission peaks for different doping concentrations in the **RB18** emitter and CBP host-based devices. The fabricated devices emitted the yellow light, with CIE coordinates from (0.47, 0.49) to (0.49, 0.49) and broad spectra from 450 to 750 nm with a maximum peak at 582 nm and color temperature range from 2671 K to 2989 K, which is a warm yellow color similar to sunset, and can be used in photo-therapeutic and comfort lighting applications.

The **RB18**-based OLEDs demonstrated driving voltages of 3.6–5.7 V, maximal current efficacies of 1.81–4.24 cd/A, maximal power efficacies of 1.29–2.91 lm/W, maximal external quantum efficiencies of 1.22–3.40% and maximal luminance in the range of 1950–2931 cd/m^2^. At higher brightness, such as 1000 cd/m^2^, which can be used for illumination applications, it was observed that the 15 wt% emitter-based device slightly outperformed the other OLEDs by displaying a maximum power efficacy of 2.91 lm/W, maximum current efficacy of 4.17 and maximal external quantum efficiency of 3.40% with a rather low driving voltage of 4.30 eV, as well as luminance of 2931 cd/m^2^.

**Figure 11:**
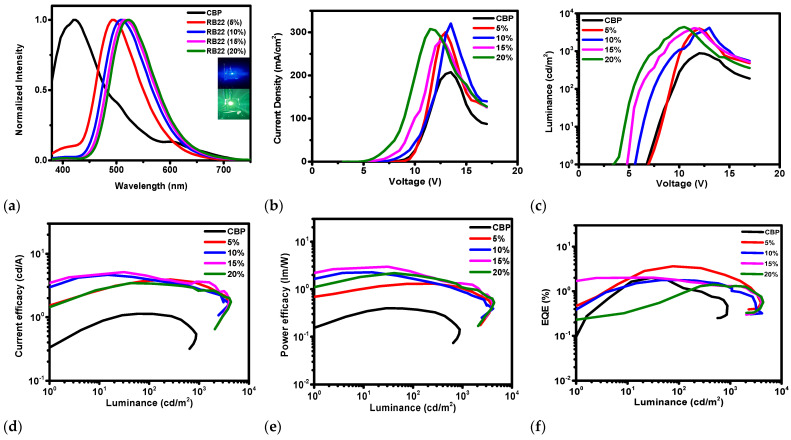
Characteristics of OLEDs using **RB22** emitter of different dopant concentrations with CBP host. (**a**) Electroluminescence spectra, Wavelength vs. Normalized intensity, (**b**) Current density–Voltage, (**c**) Luminance–Voltage, (**d**) Current efficacy–Luminance, (**e**) Power efficacy–Luminance, (**f**) External Quantum Efficiency–Luminance.

The electroluminescent (EL) properties of the devices using the emitter **RB22** at dopant concentrations of 5.0, 10.0, 15.0, and 20.0 wt% with a CBP host were also investigated. Figure 11 shows electroluminescence spectra, current density–voltage, luminance–voltage, current efficacy–luminance, power efficacy–luminance, and external quantum efficiency–luminance characteristics of the OLEDs with the emitter **RB22** dispersed in the CBP host. Some characteristics of these devices are also summarized in Table 3. The observed EL spectra (Figure 11a) showed emission peaks of devices for different doping concentrations of the **RB22** emitter in CBP. The fabricated devices emitted the green light with CIE coordinates from (0.22, 0.40) to (0.30, 0.55) and with broad spectra from 430 to 650 nm and a maximum peak at 495 nm.

The **RB22**-based OLEDs demonstrated driving voltages of 4.5–8.1 V, maximal current efficacies of 3.40–5.20 cd/A, maximal power efficacies of 1.31–2.98 lm/W, maximal external quantum efficiencies of 1.40–4.02% and maximal luminance in the range of 3986–4375 cd/m^2^. At higher brightness, such as 1000 cd/m^2^, which can be used for illumination applications, it could be stated that the 5 wt% emitter-based device slightly outperformed the other OLEDs by displaying a maximum power efficacy of 1.31 lm/W, maximum current efficacy of 3.90, and maximal external quantum efficiency of 4.02% with a rather high driving voltage of 8.10 eV, as well as luminance of 3986 cd/m^2^.

### 2.5. CIE-Chromaticity Diagram and Color Temperature

**Figure 12:**
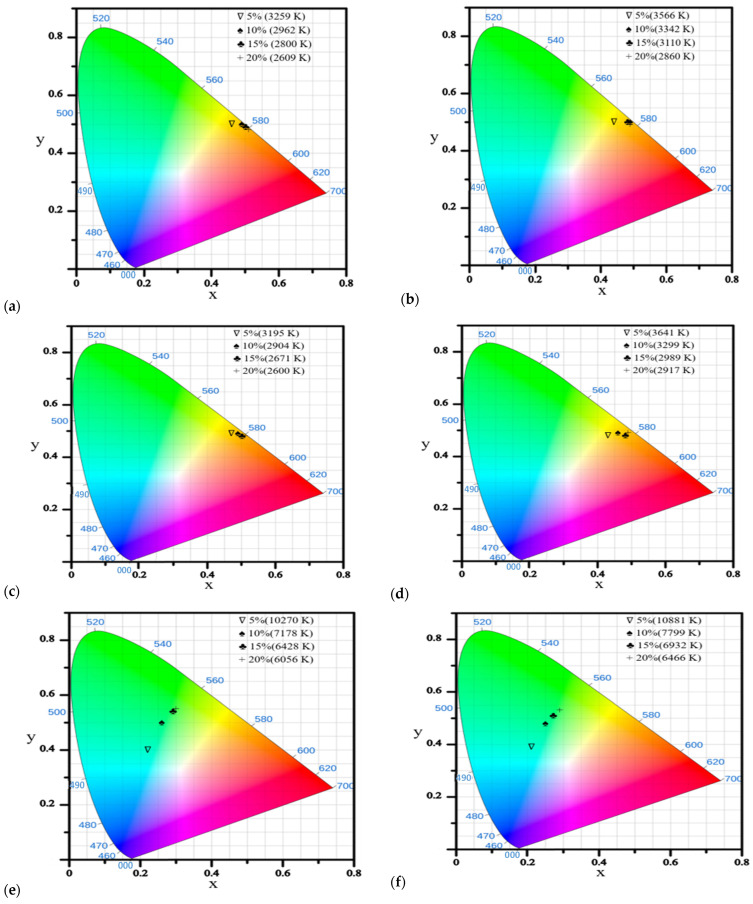
CIE-Chromaticity diagrams for these three RB materials at two different luminance levels, **RB14** (**a**) at 100 cd/m^2^, (**b**) at 1000 cd/m^2^, **RB18** (**c**) at 100 cd/m^2^, (**d**) at 1000 cd/m^2^, and **RB22** (**e**) at 100 cd/m^2^, (**f**) at 1000 cd/m^2^.

CIE-chromaticity diagrams and color temperatures are shown in Figure 12 for these devices using RB emitters with 5 to 20% doping in the CBP host matrix. Due to their color temperature range of 2600–3641 K, **RB14** and **RB18** indicated their suitability for warm lighting applications.

## 3. Experimental Part

### 3.1. Synthesis

Detailed synthesis process of the objective materials *N*-(9-ethylcarbazole-3-yl)-4-[3-(diphenylamino)phenylcarbazol-9-yl]-1,8-naphthalimide (**RB14**), *N*-phenyl-4-[3-(diphenylamino)phenylcarbazol-9-yl]-1,8-naphthalimide (**RB18**), and *N*-phenyl-4-[3-(2-methoxypyridin-3-yl)carbazol-9-yl]-1,8-naphthalimide (**RB22**) are described in experimental Section S1.1 of the Supporting Information.

### 3.2. Instrumentation

Thermogravimetric analysis (TGA) was performed on a TGAQ50 apparatus (Verder Scientific Haan, Haan, Germany). The TGA and DSC curves were recorded in a nitrogen atmosphere at a heating rate of 10 °C/min. Differential scanning calorimetry (DSC) measurements were carried out using a Bruker Reflex II thermos-system (Bruker, Berlin, Germany). The OLED devices were formed on pre-patterned ITO glass substrates, which were first cleaned with a soap solution for 10 min and then rinsed for 5 min with distilled water. Then the substrates were ultrasonically cleaned for 30 min in acetone at 50 °C and afterward for a further 30 min in isopropyl alcohol at 60 °C. After the cleaning, the substrates were treated for 15–20 min with UV to remove the solvents, and then they were transferred to a nitrogen-filled glove box. Deposition of a multilayer OLED structure was carried out in the glove box under an inert atmosphere. Hole-injecting layer of PEDOT: PSS was spin-coated at 4000 rpm for 20 s, and then the substrates were heated at 130 °C for 10 min and then cooled. An emissive layer was then spin-coated from a solution of CBP host with the dopant RB materials on the cooled substrates at 2500 rpm for 20 s. The substrates were then transferred to a thermal evaporation chamber for deposition of TPBi electron transporting and LiF injecting layer, as well as Al cathode at a high vacuum of 10^6^ torr. The device area was 0.09 cm^2^.

Characterization of the OLED devices was carried out in a completely dark room under ambient conditions. The current density–voltage–luminance characteristics were recorded using a CS-100A luminescence spectrophotometer (Becker & Hickl, Berlin, Germany), while power efficacy–luminance–current characteristics were recorded using a PR-655 spectrophotometer (NLIR, Farum, Denmark). The Keithley 2400 voltmeter (Keithley Instruments, Cleveland, OH, USA) was used to measure the current-voltage (I-V) characteristics. The external quantum efficiency (EQE) of the devices was calculated using the method described in the literature [33].

## 4. Conclusions

Three new bipolar emitting materials, **RB14**, **RB18**, and **RB22**, were synthesized and exhibited excellent thermal and electroluminescent properties. **RB18** was characterized as a bipolar OLED emitter having CIE coordinates of (0.47, 0.49) to (0.49, 0.49) and broad spectra from 450 to 750 nm with a maximum peak at 582 nm and with a color temperature range from 2671 K to 2989 K, which is a warm yellow color. This sunset-like color can be used in photo-therapeutic and comfort lighting applications. This material demonstrated thermal durability (T_d_) up to 431 °C and a high glass transition temperature (T_g_) of 162 °C, which is the highest T_g_ reported for naphthalimide-based bipolar emitter derivatives for a yellow OLEDs to date, making it suitable for photo-therapeutic and comfort lighting applications. **RB14** and **RB22** are yellowish-green and green bipolar emitters and also exhibited high T_g_ and T_d_ values, confirming their morphological and operational stability. In **RB22**, a blue shift due to H-aggregate formation causes an increase in thermal stability, which is validated through TGA, DSC, and variable-temperature UV–visible spectroscopy. **RB22** is the most thermally stable material in the group. OLED devices fabricated with a CBP host achieved maximum EQEs of 7.93% with **RB14**, 3.40% with **RB18**, and 4.02% with **RB22**, outperforming previously reported naphthalimide-based bipolar emitters. Although these emitters possess excellent intrinsic stability, further optimization of device architecture, charge-transporting layers, and fabrication processes could further improve performance. These results indicate strong potential for the development of next-generation high-efficiency, thermally robust OLEDs.

## Figures and Tables

**Table 1:** Characteristics of OLEDs using emitter **RB14** with different dopant concentrations and CBP host.

Host	Dopant Emitters		Driving Voltage (V)	Power Efficacy (PE) (lm/W)	Current Efficacy (CE) (cd/A)	EQE (%)	CIE (x, y)	Maximum Luminance (cd/m^2^)
	Materials (RB)	Conc. (wt. %)						
				@ Max [PE, CE and EQE (%)]/100 cd/m^2^/1000 cd/m^2^			at 100 and 1000 cd/m^2^	
		0	7.90	0.40/ 0.40	1.14/ 1.10	2.46/ 0.90	(0.25, 0.27)/-	878
CBP	RB14	5	5.80	2.93/ 1.50/ 0.90	5.89/ 3.60/ 2.90	4.18/ 1.30/ 1.10	(0.46, 0.50)/ (0.44, 0.50)	3722
		10	4.60	2.40/ 1.70/ 0.90	5.09/ 3.20/ 2.50	7.93/ 1.30/ 1.30	(0.49, 0.50)/ (0.47, 0.50)	3799
		15	4	2.90/ 2.90/ 1.10	7.39/ 4.70/ 2.60	4.42/ 1.80/ 1.10	(0.50, 0.49)/ (0.48, 0.50)	3746
		20	3.60	2.17/ 1.70/ 0.70	2.77/ 2.50/ 1.60	1.60/ 1.10/ 1.10	(0.51, 0.48)/ (0.49, 0.49)	3999

**Table 2:** Characteristics of OLEDs using emitter **RB18** with different dopant concentrations and CBP host.

Host	Dopant Emitters		Driving Voltage (V)	Power Efficacy (PE) (lm/W)	Current Efficacy (CE) (cd/A)	EQE (%)	CIE (x, y)	Maximum Luminance (cd/m^2^)
	Materials (RB)	Conc. (wt. %)						
				@ Max [PE, CE and EQE (%)]/100 cd/m^2^/1000 cd/m^2^			at 100 and 1000 cd/m^2^	
		0	7.90	0.40/ 0.40	1.14/ 1.10	2.46/ 0.90	(0.25, 0.27)/-	878
CBP	RB18	5	5.70	1.29/ 1.20/ 0.70	3.39/ 3.20/ 2.20	1.65/ 1.20/ 0.80	(0.47, 0.49)/ (0.43, 0.48)	2244
		10	4.90	2.60/ 1.70/ 0.70	4.24/ 3.50/ 2	2.33/ 1.40/ 0.70	(0.49, 0.49)/ (0.46, 0.49)	2338
		15	4.30	2.91/ 1.70/ 0.40	4.17/ 3.20/ 1.30	3.40/ 1.30/ 0.60	(0.50, 0.48)/ (0.48, 0.48)	2931
		20	3.60	1.38/ 1.20/ 0.50	1.81/ 1.80/ 1.20	1.22/ 0.70/ 0.50	(0.51, 0.48)/ (0.49, 0.49)	1950

**Table 3:** Characteristics of OLEDs using emitter **RB22** with different dopant concentrations and CBP host.

Host	Dopant Emitters		Driving Voltage (V)	Power Efficacy (PE) (lm/W)	Current Efficacy (CE) (cd/A)	EQE (%)	CIE (x, y)	Maximum Luminance (cd/m^2^)
	Materials (RB)	Conc. (wt.%)						
				@ Max [PE, CE and EQE (%)]/100 cd/m^2^/1000 cd/m^2^			at 100 and 1000 cd/m^2^	
		0	7.90	0.40/ 0.40	1.14/ 1.10	2.46/ 0.90	(0.25, 0.27)/-	878
CBP	RB22	5	8.10	1.31/ 1.30/ 1.10	3.90/ 3.80/ 3.50	4.02/ 2.30/ 1.80	(0.22, 0.40)/ (0.21, 0.39)	3986
		10	6.30	2.30/ 1.70/ 0.90	4.66/ 3.90/ 2.80	1.84/ 1.40/ 1	(0.26, 0.50)/ (0.25, 0.48)	4165
		15	5.10	2.98/ 2.20/ 1.30	5.20/ 4.30/ 3.40	2.05/ 1.40/ 1.20	(0.29, 0.54)/ (0.27, 0.51)	4100
		20	4.50	2.13/ 2/ 1.10	3.40/ 3.40/ 2.60	1.40/ 1.20/ 0.90	(0.30, 0.55)/ (0.29, 0.53)	4375

## Data Availability

All data produced or examined in this study are provided within this published article.

## Supplementary Materials

The following supporting information can be downloaded at: https://www.mdpi.com/article/10.3390/molecules31010158/s1. Refs. [26,27,34,35] are cited in Supplementary Materials.

## References

[1] Liguori R., Nunziata F., Aprano S., Maglione M.G. (2024). Overcoming challenges in OLED technology for lighting solutions. Electronics.

[2] Vijayaraghavan P., Liu C.H., Vankayala R., Chiang C.S., Hwang K.C. (2014). Designing multi-branched gold nanoechinus for NIR light-activated dual modal photodynamic and photothermal therapy in the second biological window. Adv. Mater..

[3] Anamika Gupta N., Sharma D., Maurya A., Kumar A., Jou J.H., Kuila B.K. (2025). Side-Chain Polarity-Dependent Photoluminescence and Deep Blue Electroluminescence in Fluorene-Based Conjugated Polymer Networks. ACS Appl. Polym. Mater..

[4] Sharma D., Gull S., Ramakrishnan A., Lenka S., Kumar A., Kumar K., Lin P.-K., Wang C.-W., Chen S.-W., Grigalevicius S. (2024). Two-Dimensional Transition Metal Dichalcogenide: Synthesis, Characterization, and Application in Candlelight OLED. Molecules.

[5] Xiang H.Y., Li Y.Q., Meng S.S., Lee C.S., Chen L.S., Tang J.X. (2018). Extremely efficient transparent flexible organic light-emitting diodes with nanostructured composite electrodes. Adv. Opt. Mater..

[6] Gu G., Forrest S.R. (2002). Design of flat-panel displays based on organic light-emitting devices. IEEE J. Sel. Top. Quantum Electron..

[7] Kim H.S., Joo C.W., Pyo B., Lee J., Suh M.C. (2017). Improvement of viewing angle dependence of the white organic light-emitting diodes with tandem structure by introduction of nanoporous polymer films. Org. Electron..

[8] Tan G., Chen S., Siu C.-H., Langlois A., Qiu Y., Fan H., Ho C.-L., Harvey P.D., Lo Y.H., Liu L. (2016). Platinum (II) cyclometallates featuring broad emission bands and their applications in color-tunable OLEDs and high color-rendering WOLEDs. J. Mater. Chem. C.

[9] Beresneviciute R., Kumar A., Blazevicius D., Lenka S., Hsieh S.-T., Tsai M.-F., Krucaite G., Tavgeniene D., Jou J.-H., Grigalevicius S. (2025). Carbazolyl Electron Donor and Pyridinyl Electron Acceptor Containing Derivatives as Potential Host Materials for Green Organic Light-Emitting Diodes. Molecules.

[10] Kanno H., Hamada Y., Takahashi H. (2004). Development of OLED with high stability and luminance efficiency by co-doping methods for full color displays. IEEE J. Sel. Top. Quantum Electron..

[11] Arias A.C., MacKenzie J.D., McCulloch I., Rivnay J., Salleo A. (2010). Materials and applications for large area electronics: Solution-based approaches. Chem. Rev..

[12] Swartwout R., Hoerantner M.T., Bulović V. (2019). Scalable deposition methods for large-area production of perovskite thin films. Energy Environ. Mater..

[13] Ho S., Liu S., Chen Y., So F. (2015). Review of recent progress in multilayer solution-processed organic light-emitting diodes. J. Photonics Energy.

[14] Lee S., Kim H., Kim Y. (2021). Progress in organic semiconducting materials with high thermal stability for organic light-emitting devices. InfoMat.

[15] Svoboda R., Krbal M. (2024). New insight into the thermal stability of the amorphous tetraphenyl-diamine (TPD)—A combined calorimetry/in-situ Raman microscopy study. Mater. Chem. Phys..

[16] Guo X., Tang Z., Yu W., Wang Y., Zhao Z., Gu J., Liu Z., Qu B., Xiao L., Chen Z. (2021). A high thermal stability terpyridine derivative as the electron transporter for long-lived green phosphorescent OLED. Org. Electron..

[17] Chesterman F., Piepers B., Kimpe T., De Visschere P., Neyts K. (2016). Influence of temperature on the steady state and transient luminance of an OLED display. J. Disp. Technol..

[18] Hua L., Liu Y., Liu B., Zhao Z., Zhang L., Yan S., Ren Z. (2022). Constructing high-efficiency orange-red thermally activated delayed fluorescence emitters by three-dimension molecular engineering. Nat. Commun..

[19] Rayappa Naveen K., Prabhu C.P.K., Braveenth R., Hyuk Kwon J. (2022). Molecular design strategy for orange red thermally activated delayed fluorescence emitters in organic light-emitting diodes (OLEDs). Chem.–Eur. J..

[20] Kagatikar S., Sunil D. (2022). A systematic review on 1, 8-naphthalimide derivatives as emissive materials in organic light-emitting diodes. J. Mater. Sci..

[21] Lenka S., Tavgeniene D., Wang H.-M., Kumar A., Lin Z.-T., Jaykumar J., Blazevicius D., Krucaite G., Grigalevicius S., Jou J.-H. (2025). Branched carbazole-based derivative is a very efficient host material for third-generation OLED devices. Synth. Met..

[22] Hasan M., Saggar S., Shukla A., Bencheikh F., Sobus J., McGregor S.K.M., Adachi C., Lo S.-C., Namdas E.B. (2022). Probing polaron-induced exciton quenching in TADF-based organic light-emitting diodes. Nat. Commun..

[23] Uoyama H., Goushi K., Shizu K., Nomura H., Adachi C. (2012). Highly efficient organic light-emitting diodes from delayed fluorescence. Nature.

[24] Mughal E.U., Naeem N., Kainat S.F., Almohyawi A.M., Qurban J., Sadiq A., Abd-El-Aziz A., Ma N., Abd-El-Aziz A.S., Timoumi A. (2025). Advances in the Design of Thermally Activated Delayed Fluorescence Materials for High-Efficiency OLEDs. J. Photochem. Photobiol. C Photochem. Rev..

[25] Liu Y., Li C., Ren Z., Yan S., Bryce M.R. (2018). All-organic thermally activated delayed fluorescence materials for organic light-emitting diodes. Nat. Rev. Mater..

[26] Tucker S.H. (1926). LXXIV.—Iodination in the carbazole series. J. Chem. Soc. (Resumed).

[27] Lennox A.J., Lloyd-Jones G.C. (2010). The Slow-Release Strategy in Suzuki-Miyaura Coupling. Isr. J. Chem..

[28] Dai Q., Liu W., Zeng L., Lee C.S., Wu J., Wang P. (2011). Aggregation-induced emission enhancement materials with large red shifts and their self-assembled crystal microstructures. CrystEngComm.

[29] Ma S., Du S., Pan G., Dai S., Xu B., Tian W. (2021). Organic molecular aggregates: From aggregation structure to emission property. Aggregate.

[30] Cravcenco A., Yu Y., Edhborg F., Goebel J.F., Takacs Z., Yang Y., Albinsson B., Börjesson K. (2021). Exciton delocalization counteracts the energy gap: A new pathway toward NIR-emissive dyes. J. Am. Chem. Soc..

[31] Samanta P.K., Saha A., Kamilya T. (2014). Chemical synthesis and optical properties of ZnO nanoparticles. Журнал нанo-та електрoннoї фізики.

[32] Zhao L.Y., Liu Y.N., Wang S.F., Tao Y.T., Wang F.F., Zhang X.W., Huang W. (2017). Novel hyperbranched polymers as host materials for green thermally activated delayed fluorescence OLEDs. Chin. J. Polym. Sci..

[33] Gautam P., Shahnawaz, Siddiqui I., Blazevicius D., Krucaite G., Tavgeniene D., Jou J.H., Grigalevicius S. (2023). Bifunctional Bicarbazole-Benzophenone-Based Twisted Donor–Acceptor–Donor Derivatives for Deep-Blue and Green OLEDs. Nanomaterials.

[34] Baneto M., Enesca A., Mihoreanu C., Lare Y., Jondo K., Napo K., Duta A. (2015). Effects of the growth temperature on the properties of spray deposited CuInS2 thin films for photovoltaic applications. Ceram. Int..

[35] Vasconcelos H.C., Meirelles M., Özmenteş R., Korkut A. (2024). Vacuum Ultraviolet Spectroscopic Analysis of Structural Phases in TiO_2_ Sol–Gel Thin Films. Coatings.

