# Supplementary material for: Thermally Stable and Energy Efficient Newly Synthesized Bipolar Emitters for Yellow and Green OLED Devices
Source: Molecules. 2026 Jan 1;31(1):158. doi: 10.3390/molecules31010158 (PMC12787045; doi:10.3390/molecules31010158)
Supplement: Supplementary file 1 [file molecules-31-00158-s001.zip › molecules-4021620-supplementary.pdf]

Thermally Stable and Energy Efficient Newly Synthesized Bipolar Emitters for Yellow and Green OLED Devices

Anil Kumar [1], Sushanta Lenka [1], Kapil Patidar [1], Chih-An Tung [1], Ming Yu Luo [1], Raminta Beresneviciute [2], Gintare Krucaite [2], Daiva Tavgeniene [2], Dovydas Blazevicius [2], Bernadeta Blazeviciute [2], Jwo-Huei Jou [1,*] and Saulius Grigalevicius [2,*]

[1] Department of Materials Science and Engineering, National Tsing Hua University, No. 101, Section 2, Guangfu Rd., East District, Hsinchu 30013, Taiwan; anilgpchkee@gmail.com (A.K.)
[2] Department of Polymer Chemistry and Technology, Kaunas University of Technology, Radvilenu Plentas 19, LT50254 Kaunas, Lithuania; dovydas.blazevicius@ktu.lt (D.B.)
* Correspondence: jjou@mx.nthu.edu.tw (J.-H.J.); saulius.grigalevicius@ktu.lt (S.G.)

Supporting file S1

1 Experimental part

1.1 Synthesis

Carbazole, 4-(diphenylamino)phenylboronic acid, 2-methoxy-3-pyridinylboronic acid, 4-bromo-1,8-naphtalic anhydride, aniline, 3-amino-9-ethylcarbazole, tetrakis(triphenylphosphine)palladium(0), bis(triphenylphosphine)palladium(II) chloride, KOH, K_2CO_3, KI, KIO_3, Na_2SO_4, acetic acid, isopropanol, chloroform, tetrahydrofuran, ethanol and dimethyl sulfoxide were purchased from Aldrich and used as received.

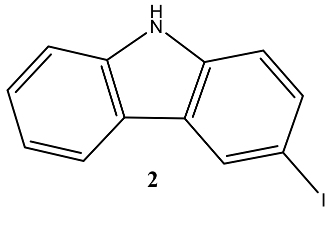

2

3-Iodo-9*H*-carbazole (**2**) was synthesized from commercially available 9*H*-carbazole by Tucker iodination with KI/KIO_3 in acetic acid [1].

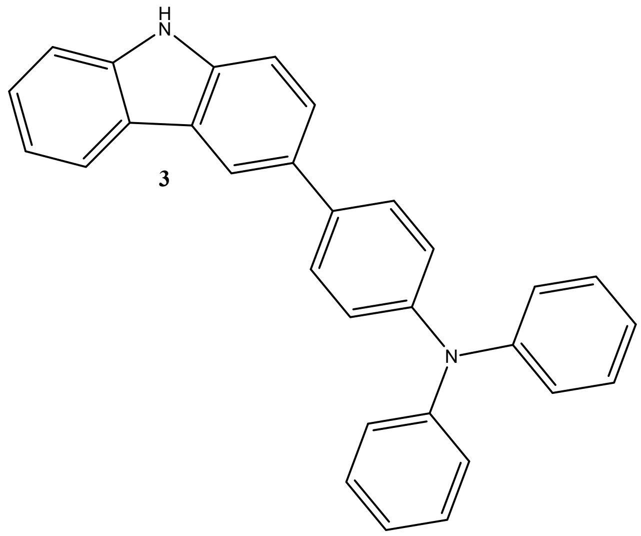

3

3-[4-(Diphenylamino)phenyl]-9*H*-carbazole (**3**). 1.00 g (3.41 mmol) of 3-iodo-9*H*-carbazole (**2**), 1.23 g (4.27 mmol) of 4-(diphenylamino)phenylboronic acid, 0.09 g (0.13 mmol) of $PdCl_2(PPh_3)_2$ and 0.95 g (16.96 mmol) of powdered potassium hydroxide were stirred in 10 ml of THF containing degassed water (1 ml) at reflux under nitrogen for 5 h. The product was extracted using chloroform after confirming the reaction progress via TLC. The combined extract was dried over anhydrous Na_2SO_4. The crude product was purified by silica gel column chromatography using the mixture of ethyl acetate and hexane (vol. ratio 1:7) as an eluent. Yield: 0.84 g of white material.

^{1}H NMR (400 MHz, $CDCl_3$-d_6, δ, m.d.): 8.33 (s, 1H, Ar), 8.17 (d, 1H, J = 8 Hz, Ar), 7.97 (s, 1H, Ar), 7.68-7.64 (m, 3H, Ar), 7.50-7.42 (m, 3H, Ar), 7.36-7.22 (m, 11H, Ar), 7.10 (t, 2H, J = 7.2 Hz, Ar). ^{13}C NMR (400 MHz, $CDCl_3$-d_6, δ, m.d.): 147.91, 146.51, 139.99, 138.77, 136.48, 132.56, 129.33, 128.01, 126.06, 125.17, 124.56, 124.25, 123.92, 123.50, 122.77, 120.43, 119.62, 118.41, 110.87, 110.81. MS (APCI+, 20 V): 411.18 ([M+H], 100 %).

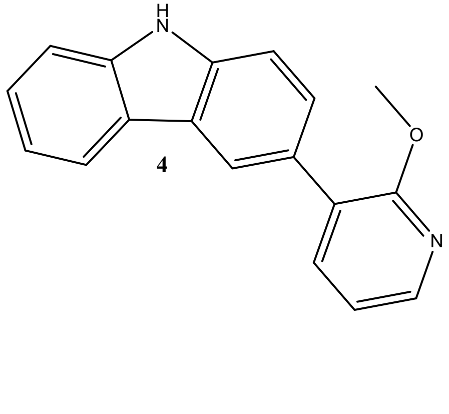

4

3-(2-methoxypyridin-3-yl)-9*H*-carbazole (**4**). 1.00 g (3.41 mmol) of 3-iodo-9*H*-carbazole (**2**), 0.67 g (4.43 mmol) of 2-methoxy-3-pyridinylboronic acid, 0.09 g (0.13 mmol) of $Pd(PPh_3)_4$, and 1.88 g (13.65 mmol) of powdered potassium carbonate were added into 10 ml of DMF and heated to reflux under a nitrogen atmosphere for 5 h. The product was extracted using chloroform after confirming the reaction completion with TLC. The combined extract was dried over anhydrous Na_2SO_4. Subsequently, the crude product was subjected to purification through silica gel column chromatography, employing a mixture of tetrahydrofuran and hexane (volume ratio 1:7) as the eluent. Yield: 0.74 g of white material.

^{1}H NMR (400 MHz, $CDCl_3$-*d*$_6$, δ, m.d.): 8.28 (s, 1H, Ar), 8.22 (s, 2H, Ar), 8.14 (d, 1H, J = 8 Hz, Ar), 7.76 (d, 1H, J = 8 Hz, Ar), 7.66 (d, 1H = J = 7.6 Hz, Ar), 7.49-7.45 (m, 3H, Ar), 7.30 (s, 1H, Ar), 7.05 (t, 1H, J = 7.4 Hz, Ar), 4.06 (s, 3H, $-CH_3$). ^{13}C NMR (400 MHz, $CDCl_3$-*d*$_6$, δ, m.d.): 161.10, 144.99, 139.90, 138.99, 138.92, 127.29, 126.02, 123.41, 121.08, 120.43, 119.60, 117.24, 100.73, 110.31, 53.70. MS (APCI+, 20 V): 275.11 ([M+H], 100 %).

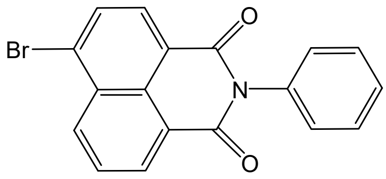

6

N-phenyl-4-bromo-1,8-naphthalimide (**6**). 1.50 g (3.61 mmol) of 4-bromo-1,8-naphthalimide (**5**) and 0.66 g (4.69 mmol) of aniline were stirred in 30 ml of ethanol. The mixture was heated to reflux for 24 h. After TLC control, the product was washed with hexane and filtered off. Yield: 1.28 g of light brown powder.

^{1}H NMR (400 MHz, $CDCl_3$-*d*$_6$, δ, m.d.): 8.62 (d, 1H, J = 7.2 Hz, Ar), 8.55 (d, 1H, J = 8.4 Hz, Ar), 8.37 (d, 1H, J = 8 Hz, Ar), 8.00 (d, 1H, J = 7.6 Hz, Ar), 7.8 (t, 1H, J = 7.6 Hz, Ar), 7.50-7.35 (m, 3H, Ar), 7.24 (d, 2H, J = 7.6 Hz, Ar). ^{13}C NMR (400 MHz, $HCDCl_3$-*d*$_6$, δ, m.d.): 163.81, 163.77, 135.11, 133.66, 132.47, 131.62, 131.24, 130.81, 130.70, 129.47, 129.38, 128.88, 128.57, 128.22, 123.26, 122.38. MS (APCI+, 20 V): 351.99 ([M+H], 100 %).

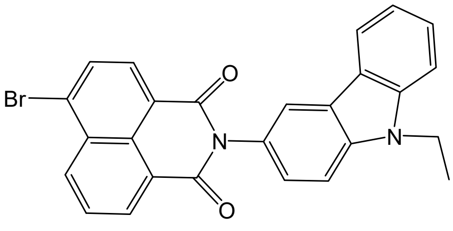

7

N-(9-Ethylcarbazol-3-yl)-4-bromo-1,8-naphthalimide (**7**) was prepared following a previously described procedure [2].

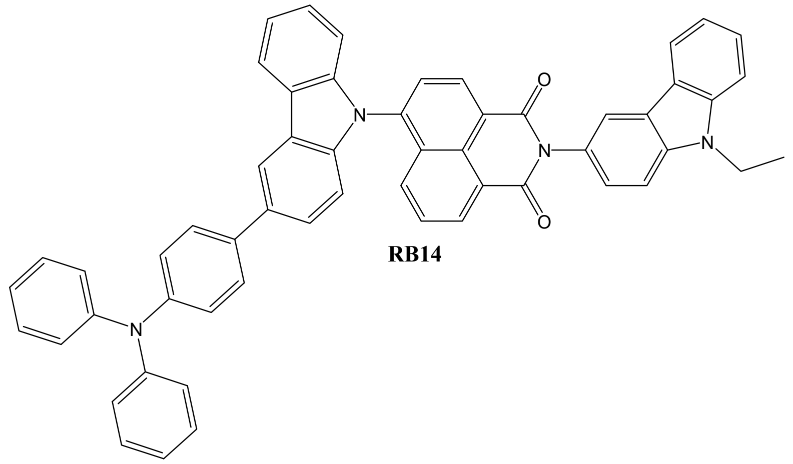

RB14

N-(9-ethylcarbazole-3-yl)-4-[3-(diphenylamino)phenylcarbazol-9-yl]-1,8-naphthalimide (**RB14**). 0.6 g (2.14 mmol) of *N*-(9-Ethylcarbazole-3-yl)-4-bromo-1,8-naphtalymide (**7**), 0.68 g (2.77 mmol) of 4–(diphenylamino)phenyl-9*H*-carbazole (**3**), and 1.76 g (21.36 mmol) of potassium carbonate were stirred in 10 ml of DMSO at 150 °C under nitrogen. The mixture was left to react for 24h. After TLC control, the inorganic materials were filtered off, and the product was extracted with chloroform. The combined extract was dried over anhydrous Na_2SO_4. The crude product was purified by silica gel column chromatography using the mixture of ethyl acetate and hexane (vol. ratio 1:7) as an eluent. Yield: 0.10 g of yellow powder.

^{1}H NMR (400 MHz, $CDCl_3$-*d*$_6$, δ, m. d.): 8.60 (d, 2H, J = 7.2 Hz, Ar), 8.19 (d, 2H, J = 8 Hz, Ar), 8.03-7.82 (m, 3H, Ar), 7.72 (t, 2H, J = 7.6 Hz, Ar), 7.62-7.28 (m, 10H, Ar), 7.22-6.94 (m, 14H, Ar), 4.35 (q,

2H, J = 7.6 Hz, $-CH_2-$), 1.40 (t, 3H, J = 7.2 Hz, $-CH_3$).^{13}C NMR (400 MHz, $CDCl_3$-*d*$_6$, δ, m. d.): 165.06, 147.78, 142.21, 140.53, 140.48, 139.71, 134.19, 132.47, 132.01, 131.79, 131.63, 129.31, 128.01, 127.05, 125.99, 125.64, 124.34, 124.30, 123.09, 122.87, 120.93, 120.81, 120.67, 119.10, 119.02, 109.25, 109.17, 108.61, 37.75, 13.95. MS (APCI+, 20 V): 798.62 ($[M]^+$, 100 %).

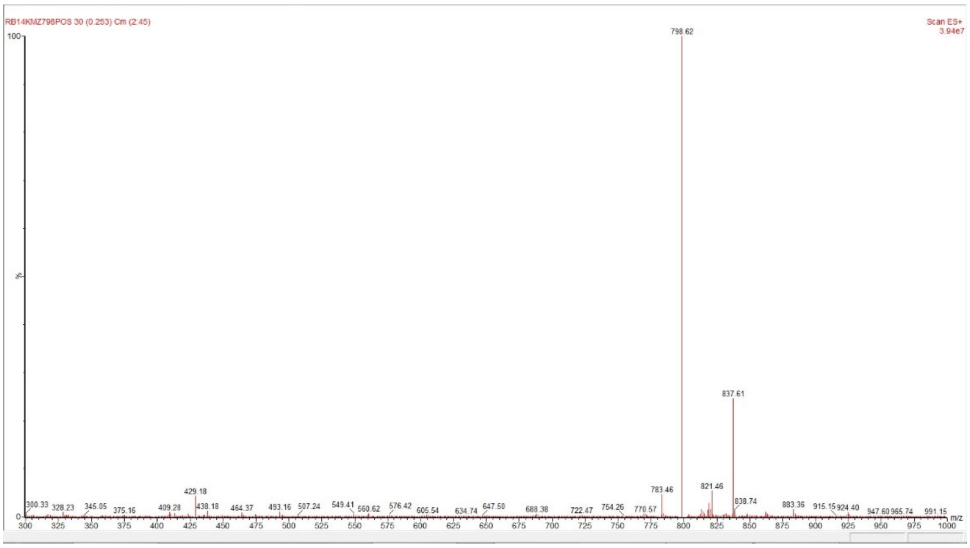

Figure S1. MS spectrum of **RB14** compound

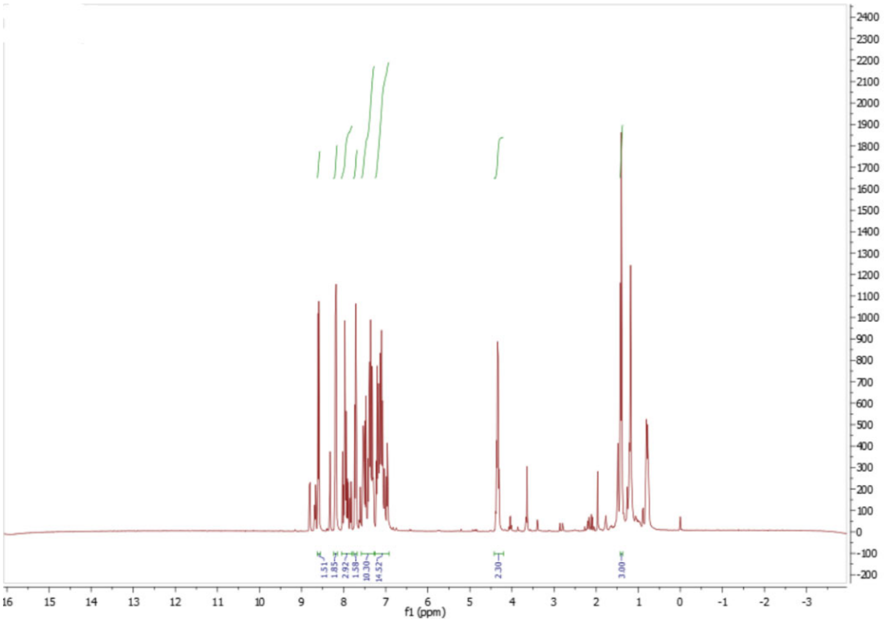

Figure S2. ^{1}H NMR spectrum of **RB14** in $CDCl_3$

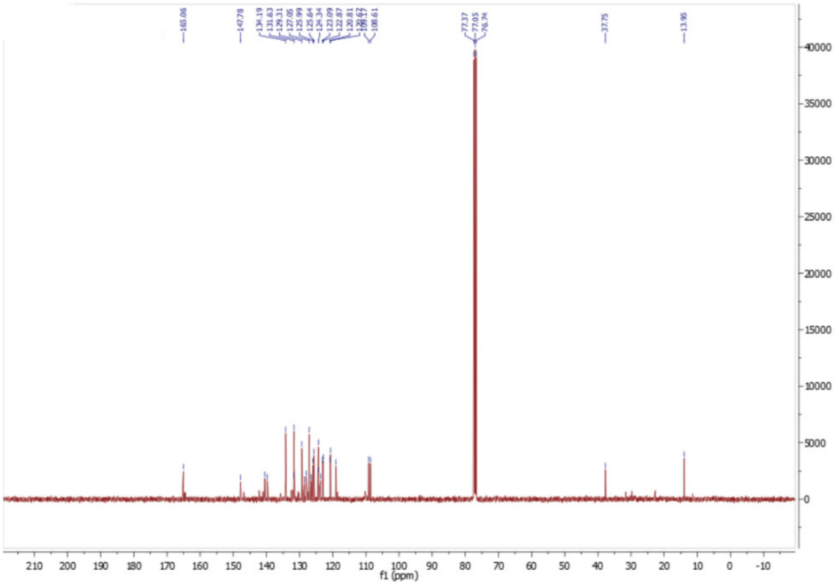

Figure S3. ^{13}C NMR spectrum of **RB14** in $CDCl_3$

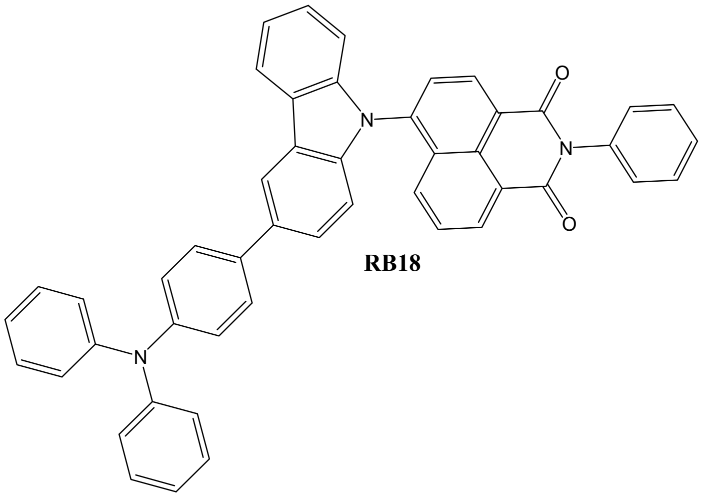

RB18

N-phenyl-4-[3-(diphenylamino)phenylcarbazol-9-yl]-1,8-naphthalimide (**RB18**). 0.50 g (4.27 mmol) of *N*-phenyl-4-bromo-1,8-naphtalymide (**6**), 0.38 g (2.84 mmol) of 4-(diphenylamino)phenyl-9*H*-carbazole (**3**), and 1.31 g (28.49 mmol) of potassium carbonate were stirred in 10 ml of DMSO at 150 °C under nitrogen. The mixture was left to react for 24h. After TLC control, the inorganic materials were filtered off, and the product was extracted with chloroform. The combined extract was dried over anhydrous Na_2SO_4. The crude product was purified by silica gel column chromatography using the mixture of ethyl acetate and hexane (vol. ratio 1:7) as an eluent. Yield: 0.19 g of yellow powder.

^{1}H NMR (400 MHz, $CDCl_3$-d_6, δ, m. d.): 8.77 (d, 1H, J = 7.6 Hz, Ar), 8.65-8.55 (m, 3H, Ar), 8.32 (s, 1H, Ar), 8.20-8.17 (m, 2H, Ar), 7.89 (d, 1H, J = 8 Hz, Ar), 7.84-7.78 (m, 2H, Ar), 7.60 (t, 1H, J = 8 Hz, Ar), 7.48-7.39 (m, 3H, Ar), 7.32-7.27 (m, 3H, Ar), 7.22-7.17 (m, 5H, Ar), 7.11-7.07 (m, 5H, Ar), 7.00-6.94 (m, 4H, Ar). ^{13}C NMR (400 MHz, $CDCl_3$-d_6, δ, m. d.): 164.14, 163.74, 147.77, 146.84, 142.18, 141.00, 140.68, 135.74, 135.25, 134.30, 134.04, 133.68, 132.48, 132.04, 131.65,130.55, 129.52, 129.47, 129.42, 129.31, 129.18, 128.92, 128.66, 128.57, 128.01, 127.80, 127.41, 126.60, 125.64, 124.41, 124.34, 124.29, 124.01, 123.62, 122.88, 120.95, 120.72, 118.64, 110.25, 110.15. MS ($APCI_+$, 20 V): 681.46 ($[M]^+$, 100 %).

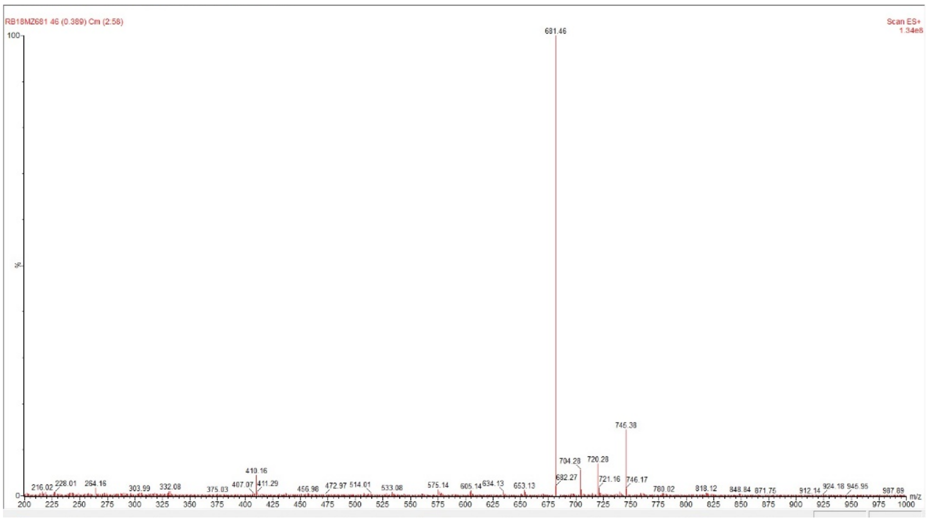

Figure S4. MS spectrum of **RB18** compound

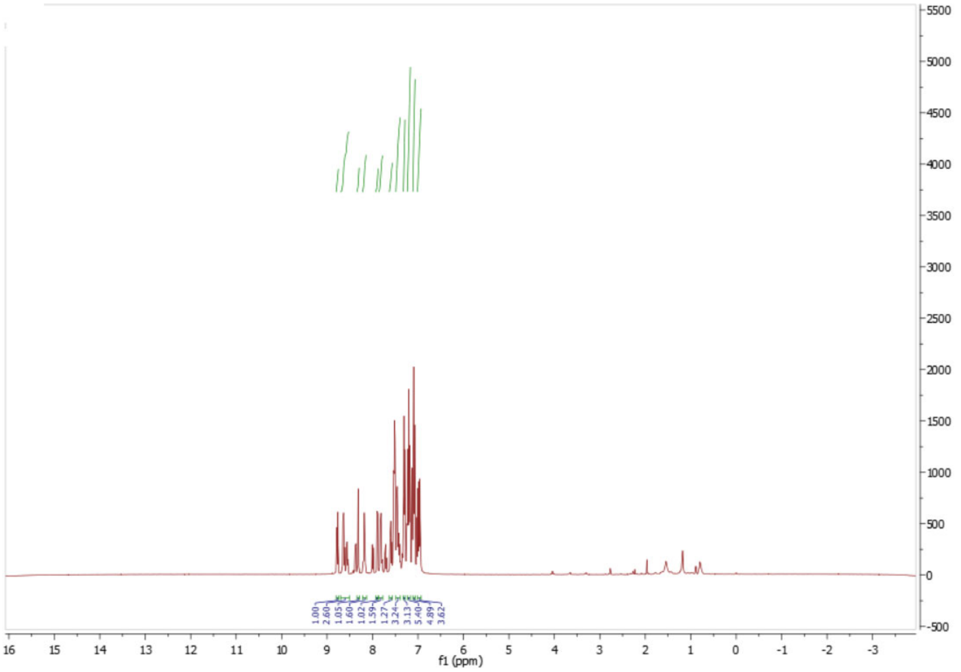

Figure S5. ^{1}H NMR spectrum of **RB18** in $CDCl_3$

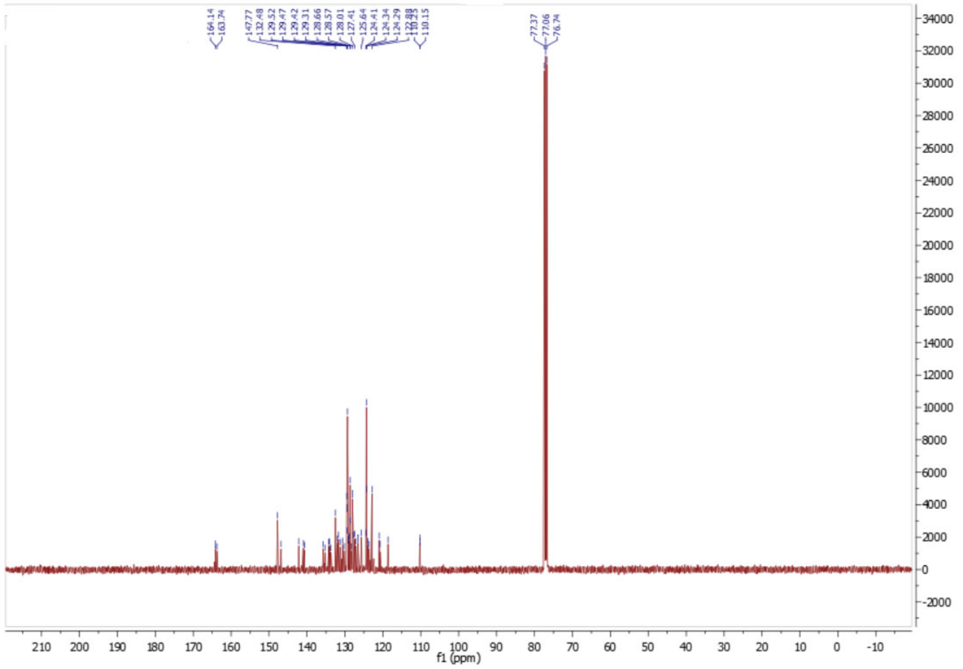

Figure S6. ^{13}C NMR spectrum of **RB18** in $CDCl_3$

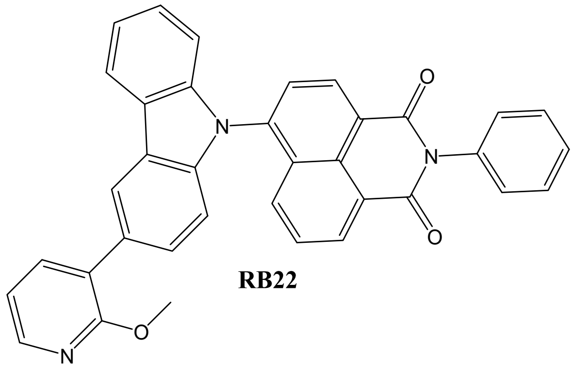

RB22

N-phenyl-4-[3-(2-methoxypyridin-3-yl)carbazol-9-yl]-1,8-naphthalimide (**RB22).** 0.19 g (4.27 mmol) of *N*-phenyl-4-bromo-1,8-naphtalymide (**6**), 0.1 g (2.85 mmol) of 3-(2-methoxypyridin-3-yl)-9*H*-carbazole (**4**), and 0.50 g (28.49 mmol) of potassium carbonate were stirred in 10 ml of DMSO at 150 °C under nitrogen. The mixture was left to react for 24h. After TLC control, the inorganic materials were filtered off, and the product was extracted with chloroform. The combined extract was dried over anhydrous Na_2SO_4. The crude product was purified by silica gel column chromatography using a mixture of ethyl acetate and hexane (vol. ratio 1:10) as an eluent. Yield: 0.12 g of yellow powder.

^{1}H NMR (400 MHz, $CDCl_3$-*d₆*, δ, m. d.): 8.88 (d, 1H, J = 8 Hz, Ar), 8.75 (d, 1H, J = 7.2 Hz, Ar), 8.42 (s, 1H, Ar), 8.28-8.22 (m, 2H, Ar), 8.00-7.93 (m, 2H, Ar), 7.77 (d, 1H, J = 7.2 Hz, Ar), 7.71 (t, 1H, J = 7.6 Hz, Ar), 7.64-7.55 (m, 4H, Ar), 7.42-7.38 (m, 4H, Ar), 7.15-7.05 (m, 3H, Ar), 4.06 (s, 3H, $-CH_3$). ^{13}C NMR (400 MHz, $CDCl_3$-d_6, δ, m. d.): 164.13, 163.73, 161.01, 145.40, 142.13, 141.17, 140.59, 138.90, 135.24, 132.50, 132.03, 130.53, 130.14, 129.55, 129.52, 129.21, 128.92, 128.65, 127.83, 127.82, 127.43, 126.61, 125.08, 123.98, 123.91, 123.62, 122.94, 121.36, 120.99, 120.77, 117.28, 110.14, 109.74, 53.74. MS ($APCI_+$, 20 V): 584.24 ($[M+K]^+$, 100 %).

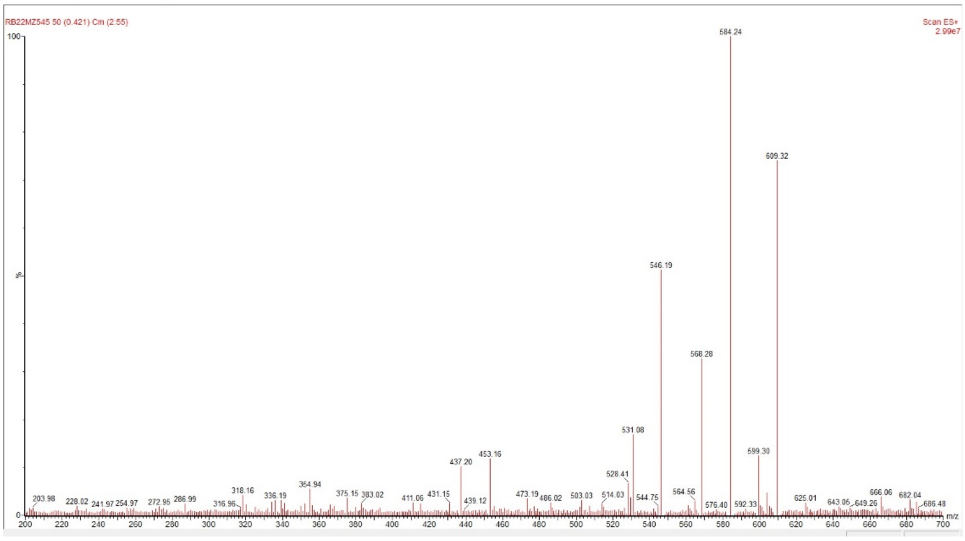

Figure S7. MS spectrum of **RB22** compound

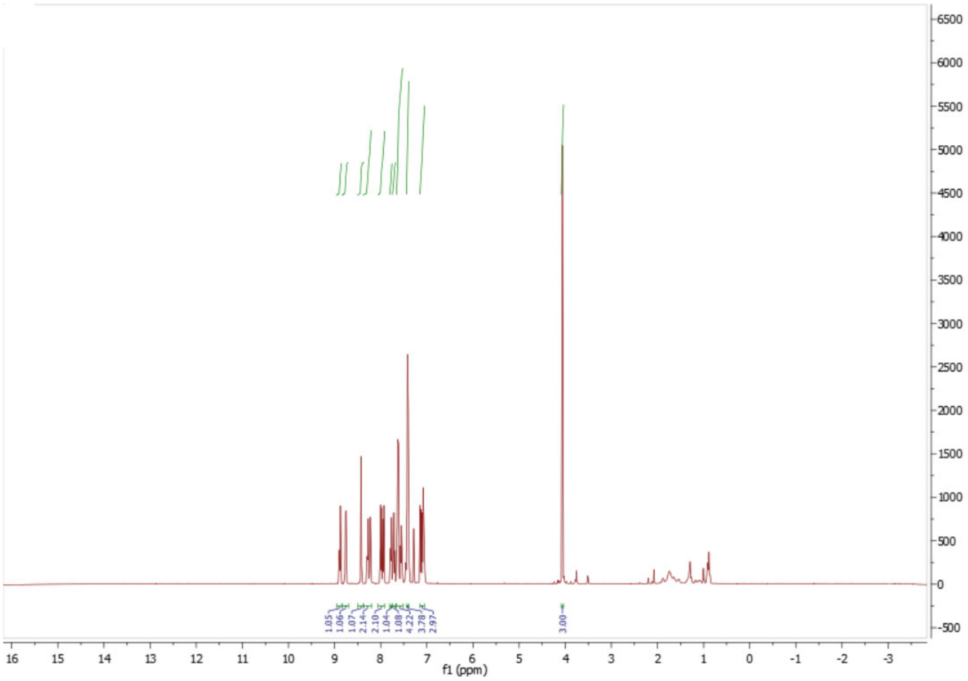

Figure S8. ^{1}H NMR spectrum of **RB22** in $CDCl_3$

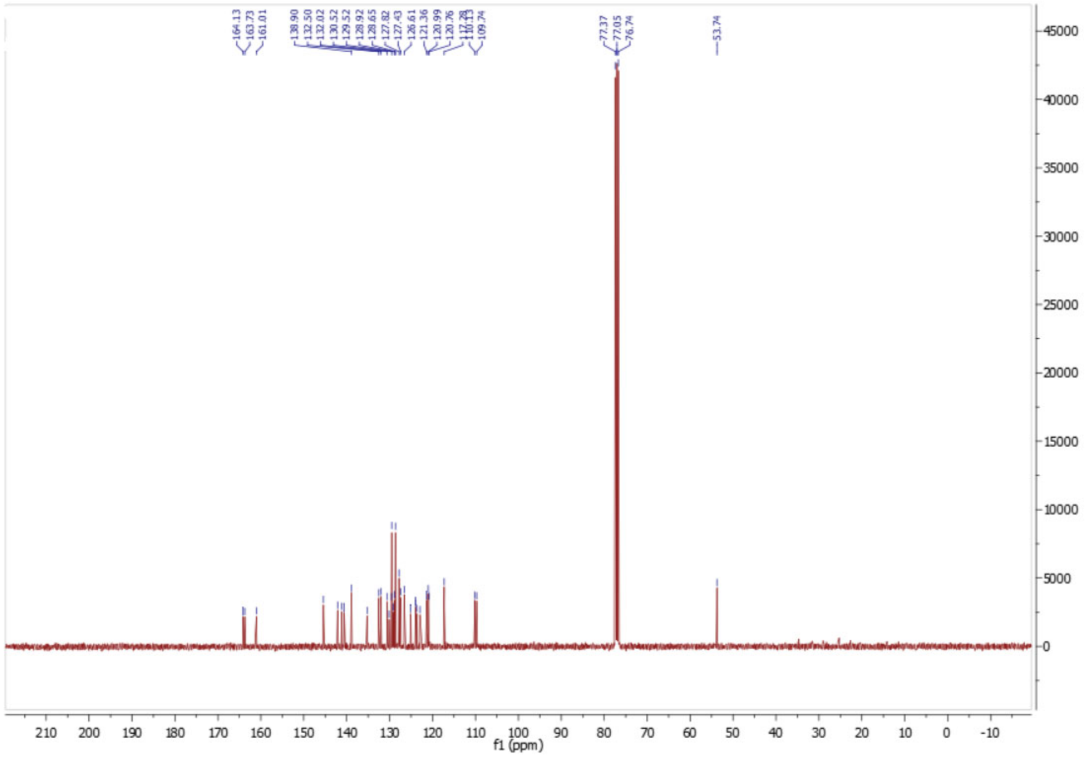

Figure S9. ^{13}C NMR spectrum of **RB22** in $CDCl_3$

1.2 UV-Visible Spectroscopy

The ultraviolet-visible absorbance (UV abs) spectra of spin-coated glass films of compounds RB14, RB18, and RB22 were examined in the temperature range from 25 °C to 200 °C for thermal stability validation, shown in Figure S1 (a–c) [3, 4]

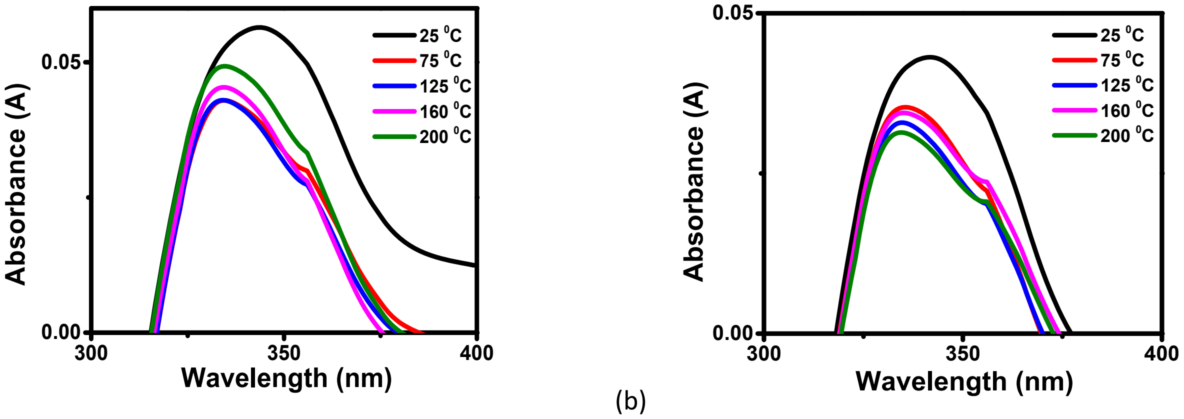

(a) (b)

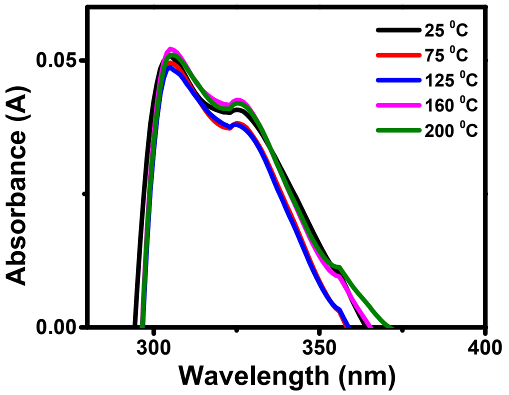

(c)

Figure S10. UV Visible Spectroscopy, (a) RB14, (b) RB18, and (c) RB22

HOMO, LUMO, and Bandgap Energy Table S1

Materials	E_{HOMO} (eV)	E_{LUMO} (eV)	Bandgap Energy (E_g= eV)
RB14	5.16	2.68	2.48
RB18	5.05	2.59	2.46
RB22	4.76	2.15	2.61

Table S2

Photo-physical Properties at Room temperature

Sample	Absorption (λ_{max}) (nm)		PL ($\lambda_{emission}$) (nm)	
	THF Solvent	Glass Films	THF Solvent	Glass Film
**RB14**	306	344	525	600
**RB18**	310	342	520	600
**RB22**	308	305	555	535

Reference

[1] Tucker, S. H. (1926). LXXIV.—Iodination in the carbazole series. *Journal of the Chemical Society (Resumed)*, *129*, 546-553.

[2] Lennox, A. J., & Lloyd-Jones, G. C. (2010). The Slow-Release Strategy in Suzuki–Miyaura Coupling. *Israel Journal of Chemistry*, *50*(5-6), 664-674.

[3] Baneto, M., Enesca, A., Mihoreanu, C., Lare, Y., Jondo, K., Napo, K., & Duta, A. (2015). Effects of the growth temperature on the properties of spray deposited CuInS2 thin films for photovoltaic applications. *Ceramics International*, *41*(3), 4742-4749.

[4] Vasconcelos, H. C., Meirelles, M., Özmenteş, R., & Korkut, A. (2024). Vacuum Ultraviolet Spectroscopic Analysis of Structural Phases in TiO2 Sol–Gel Thin Films. *Coatings*, *15*(1), 19.
